# Magnetic susceptibility in the assessment of toxic heavy metal elements in the surface sediments of Inner Ambon Bay, Maluku province, Indonesia

**DOI:** 10.1016/j.heliyon.2024.e27497

**Published:** 2024-03-11

**Authors:** Yohansli Noya, Satria Bijaksana, Silvia Jannatul Fajar, Putu Billy Suryanata, Ulvienin Harlianti, Khalil Ibrahim, Ni Komang Tri Suandayani, Warni Multi, Samsul Bahri

**Affiliations:** a Institut Teknologi Bandung, Indonesia; b Udayana University, Bali, Indonesia; c Pattimura University, Indonesia

**Keywords:** Inner Ambon bay, Surface sediments, Magnetic susceptibility, Heavy metals, Ecological risk

## Abstract

The Inner Ambon Bay (IAB) is an important area for the economic development of the city of Ambon, one of only a few urban areas in eastern Indonesia. This study is intended to monitor the anthropogenic impact on IAB by employing combined rock magnetic and geochemical analyses on 20 samples collected from IAB and the surrounding rivers. Magnetic susceptibility values of samples in the IAB averaged 26.37× 10^−8^ m^3^/kg, which is relatively high and comparable to that of contaminated coastal environments. Magnetic susceptibility correlated positively with certain metals such as Cr, Co, Ni, and Mn but negatively with Hg. Geochemical analyses showed that Hg and Ag contents were relatively high but pose only moderate risk to the environment based on the geo-accumulation index. Furthermore, the potential ecological risk index (*PERI*) showed that there were two points that showed moderate ecological risk. Multivariate statistical analysis (principal component analysis (PCA), Pearson's correlation coefficient (PCC), and hierarchical cluster analysis (HCA)) outlined that the metallic accumulation in the sediments of IAB was related to lithological, geological, and anthropogenic impacts. Therefore, oil spills and household waste are likely major reasons for anthropogenic pollution in the sediments of the IAB.

## 1 Introduction

The United Nations (UN) has outlined seventeen Sustainable Development Goals (SDGs), one of which is Goal 11: Sustainable Cities and Communities, aiming to make urban areas inclusive, safe, resilient, and sustainable (https://sdgs.un.org/goals). One of the challenges faced by urban areas is pollution resulting from the significant increase in urban population activities. This challenge is also experienced in urban areas in Indonesia. One way to address this challenge is to monitor the levels or concentrations of anthropogenic components released or discharged into the environment, including aquatic environments (rivers, lakes, and coastal areas).

In Indonesia, the impact of anthropogenic components on aquatic environments is assessed by various methods, including a combination of rock magnetic and geochemical analyses. Previously, such combined methods were used in analyzing leachate muds in waste disposal sites in West Java [1,2], lake sediments in Gorontalo [3,4], as well as sediments in various rivers such as the Citarum River in West Java [5], the Brantas River in East Java [6,7], the Krueng Aceh River in Aceh [8], and the rivers in Kalimantan Selatan [9].

In other countries, such combined methods have been used successfully to analyze pollution in coastal areas. The pioneering work of Chan et al. [10] in Penny's Bay in Hong Kong found that contaminated sediments had significantly higher magnetic susceptibility compared to uncontaminated sediments. They also found a positive correlation between magnetic susceptibility and the contents of Pb, Zn, and Cu. Similar studies were conducted in many coastal areas, such as the Bay of Bengal [11], the coast of Kerala [12], the Shilaorem Beach [13], the coast of Fujian [14], the Zhousan Island [15], and the Richards Bay Harbor, South Africa [16]. Similar studies have also been conducted on sediments in the Asopos River and its estuary in the South Evoikos Gulf, Greece [17] and in Vembanad Lagoon in southwestern India [18]. In volcanic islands such as Indonesia, magnetic minerals, which are considered heavy metal elements elsewhere, might originate from natural rocks in the surrounding environment [5].

Ambon Bay, an estuarine bay located in eastern Indonesia, serves as a vital hub for fisheries cultivation, tourism, and navigation. At the core of economic operations, Ambon Port stands as a crucial focal point. However, the influx of substantial waste into the bay exerts significant pressure on its aquatic ecosystem. Additional sources of metal pollution stem from domestic and urban areas, as well as fishing boat coatings and agricultural runoff. Furthermore, tourism activities may contribute to heavy metal leaching, posing a potential hazard to the ecological balance of Ambon Bay. Therefore, it is important to improve our understanding of the current levels of heavy metal concentrations in the sediments of these coastal environments. The study area, Inner Ambon Bay (IAB), is a prospective site for monitoring anthropogenic impacts on coastal areas in Indonesia, which is the largest archipelago in the world. This study is intended to serve as a test of the effectiveness of combined rock magnetic and geochemical analyses in monitoring anthropogenic pollutants in marine environments in tropical regions with complex geological conditions, such as Indonesia.

## 2 Materials and methods

### 2.1 Study area and sample collection

**Fig. 1:**
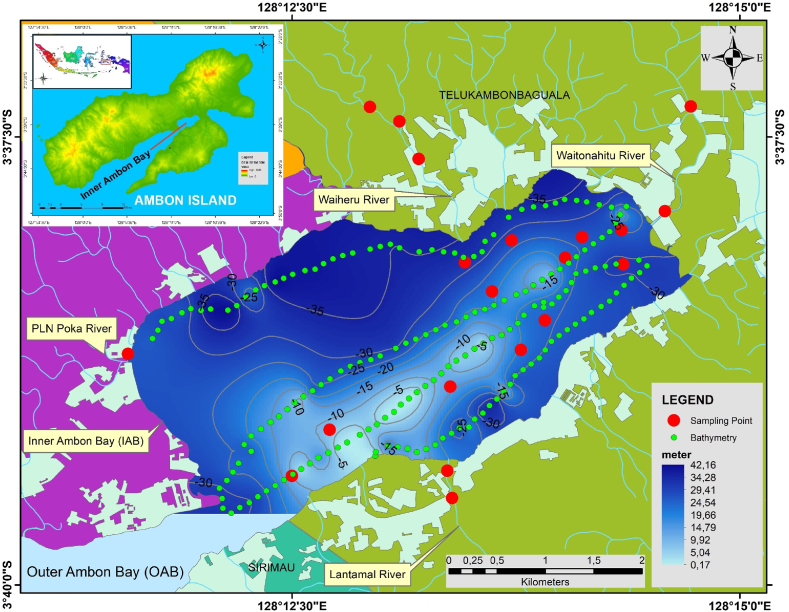
Location of the study area (Inner Ambon Bay) and the sampling site.

The IAB is a part of Ambon Bay that is connected to the Banda Sea. Since 2016, the suspended *Jembatan Merah Putih* (the Red and White Bridge) has been inaugurated and has served as the boundary between IAB and the Outer Ambon Bay (UAB). The IAB, with an approximate area of 11 km2 [19], and based on our bathymetric measurements, has a known depth ranging from 0.17 to 42.16 m, receives surface runoff from Ambon City through three main rivers (Waitonahitu River, Waiheru River, and Lantamal River), as well as smaller rivers and water channels. In addition to runoff from Ambon City, there are also the Halong Ferry Port and the Galala Fishing Port, which may contribute to anthropogenic components in IAB sediments. Fig. 1 shows the location of IAB. Earlier, the pollution level and waste concentration in the IAB were studied [19,20], particularly in the context of utilizing the IAB for economic activities such as fishing (floating net cage), ports, and tourism.

**Fig. 2:**
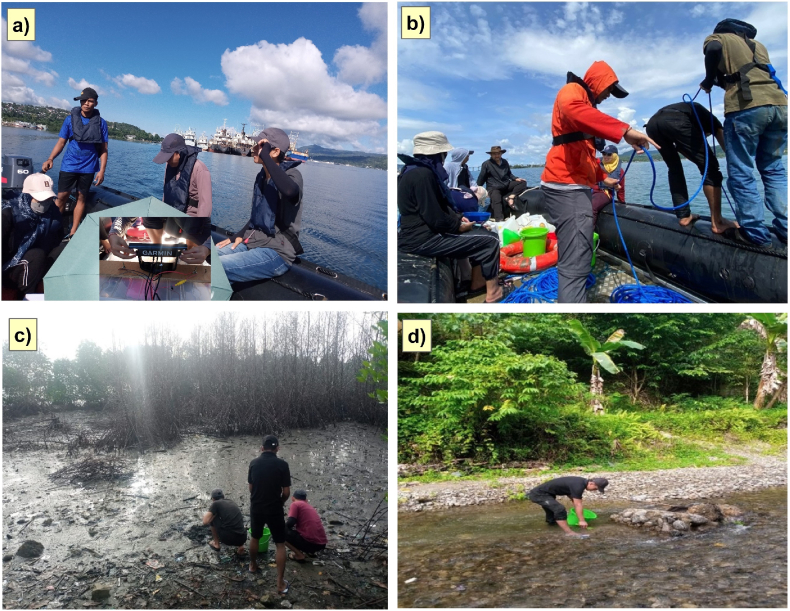
a) Determining the water depth using Garmin Striker Plus 7SV APAC equipment; b) Lowering the sediment grabber with a rope to collect sediments from Inner Ambon Bay (IAB); c) The location of KL01 in PLN Poka River, with moderate potential ecological risk; as a result, the mangrove forests dry up and die. d) Samples are taken from the downstream, middle, and upstream of the Waiheru River, Waitonahitu River, PLN Poka River, and Lantamal River.

Fieldwork activities were conducted from March to April 2023, which included collecting sediment samples from the IAB area and sediment samples from rivers that flow into the IAB. Fig. 1 shows the locations of the sediment sample collection points in the IAB. Fig. 2 illustrates the surveys conducted in this study. The sample collection positions were determined using GPS equipment, while water depth was measured using Garmin Striker Plus 7SV APAC equipment (Fig. 2a). Sediment sample collection (Fig. 2b) was conducted using a sediment grabber tool attached to a boat in the IAB. There are a total of 12 sediment sampling points in the IAB (Fig. 2b and c) and 8 sediment sampling points in rivers that flow into the IAB (Fig. 2d).

### 2.2 Analytical methods

Sediment sample preparation involves adding 10 ml of nitric acid (HNO_3_) to every 5 L of sediment sample obtained [21]. The aim is to clean the sample from organic contaminants and dissolve heavy metals bound to the sediment matrix, thereby preparing the sample for more accurate laboratory analysis. Subsequently, the samples were prepared for magnetic and geochemical analyses. The sediment samples were sieved and air-dried at room temperature. The sediment samples obtained were sieved through a 325-mesh sieve. The dried samples were also placed in cylindrical plastic containers with a diameter of 25.4 mm and a height of 22 mm (with a volume of 10 cm^3^). These samples were used for magnetic susceptibility measurements using the Bartington MS3 magnetic susceptibility system (Bartington Instruments Ltd., Witney, UK), which measures mass-based low-frequency magnetic susceptibility (χ_*LF*_) and mass-based high-frequency magnetic susceptibility (χ_*HF*_) using the MS2B sensor operating at respective frequencies of 470 Hz and 4700 Hz.

To determine toxic heavy metal concentrations in the sediment samples, it is usually necessary to extract the absorbed metals and then analyze them using procedures such as atomic absorption spectrometry (AAS) or inductively coupled plasma mass spectrometry (ICP-MS). However, the process of extracting and analyzing resin is known for being slow, expensive, and time-consuming. To overcome these constraints, EDAX Orbis Micro X-ray Fluorescence Spectrometry (μ-XRF) was used due to its fast, non-destructive, high-resolution, and multi-element capabilities across various fields of application, including materials science, quality control, environmental science, geology, and archaeology [22,23]. Micro XRF (μ-XRF) equipment offers better spatial resolution than standard XRF equipment [24]. A Rhodium X-ray source-equipped portable ED-XRF analyzer was employed to conduct elemental analysis on homogenized fine fraction seaweed samples. The sample, representative in nature, was positioned centrally above the detector window, which had an active surface of 20 mm^2^. For the analysis of macroelements, the GeoMining calibration mode was chosen, involving three-phase measurements, each lasting 100 s. The voltage and current applied in these phases were 40 kV and 355 μA, respectively. Bruker's Instrument Tools (v. 1.6.0.110) were utilized to process the raw spectral data. To derive quantitative data, integral spectra were exported through the Bruker Artax software (v. 8.0.0.476).

### 2.3 Data analysis

To find out where the pollution comes from and how dangerous it is for the environment, background values, or local baselines (Bn), for heavy metals and REEs in nature were chosen based on previous research. This is because the amount of metal pollution in sediments will show how polluted they are. Two background values were used for different environments. For instance, for Pb, two different values were used, i.e., 29 for marine and 20 for river, knowing that geochemical values are different between rivers and seas. For some elements, the background values for river and marine environments were similar. The background values for various elements in milligrams per kilogram (mg/kg) were as follows: Pb (29 and 20), Zn (114 and 95), Cu (29 and 45), As (13), Cd (0.26 and 0.3), Hg (0.4), Cr (90), Co (19), Ni (68), Ag (0.07), Sb (1.5), Ti (10), Mn (850), Fe (3600), Gd (14.82), Nd (2.59), La (2.02) [[25], [26], [27]]. The spatial distribution of single toxic heavy metals, REEs, and potential ecological risk to the environment by multiple pollutants of each content in sediments was analyzed by ArcGIS Pro 2.6.3, using the Kriging grid method. Statistical analyses were conducted in MS Excel 2019 and Origin 2023b, which were employed for Pearson correlation coefficient, principal component analysis (PCA), and hierarchical cluster analysis (HCA), respectively. These statistical approaches facilitated the evaluation of potential sources and relationships among toxic heavy metal elements present in the surface sediments of the study area [28,29]. The appropriateness of the figures for PCA was thoroughly confirmed through Bartlett's sphericity tests, helps clarify whether the data sets for the subjects are suitable for analysis [29].

### 2.4 Magnetic susceptibility

Apart from $\chi_{LF}$ and $\chi_{HF}$, another term, frequency-dependent magnetic susceptibility or $\chi_{FD\%}$ was used as defined in the following equation:

$$\chi_{FD\%}=100\;\%\times \frac{\left(\chi_{LF}-\chi_{HF}\right)}{\chi_{LF}}$$

The parameter $\chi_{LF}$ is often used as a proxy indicator for the concentration of magnetic minerals, especially ferromagnetic mineral phases like magnetite and hematite [3,5], while the parameter $\chi_{FD\%}$ is frequently used to determine the concentration of superparamagnetic (SP) fine grains in samples. A higher $\chi_{FD\%}$ value indicates a higher concentration of SP grains, and vice versa [1,30].

### 2.5 Geo-accumulation index (I_geo_)

The geo-accumulation index (*I*_*geo*_) used to evaluate heavy metal and REE pollution in sediments was introduced by Müller and is identified by the following equation [31]:

$$I_{geo}=\log_{2}\left(\frac{C_{n}}{1.5\times B_{n}}\right)$$

where *C*_*n*_ (mg/kg) is the measured concentration of the metal and REE, and *B*_*n*_ (mg/kg) is the background concentration of the metal and REE. A factor of 1.5 is the background matrix correction factor due to the lithogenic effects. A positive *Igeo* index value for heavy metals signifies the existence of anthropogenic sources of some toxic heavy metals, while a negative *Igeo* index value for heavy metals does not indicate the presence of anthropogenic sources [32]. The degree of heavy metal contamination (grades of *I*_*geo*_) is categorized in Förstner et al. [33] and represented in Table 1.

**Table 1:** The following seven-level classification of I_geo_ is given by Förstner et al. (1990).

Class	Value	Level of contamination classification
0	I_geo_ ≤ 0	Uncontaminated
1	0 < I_geo_ < 1	Uncontaminated to moderately contaminated
2	1 < I_geo_ < 2	Moderately contaminated
3	2 < I_geo_ < 3	Moderately to heavily contaminated
4	3 < I_geo_ < 4	Heavily contaminated
5	4 < I_geo_ < 5	Heavily to extremely contaminated
6	5 < I_geo_	Extremely contaminated

### 2.6 Potential ecological risk index (PERI)

The potential ecological risk index (*PERI*) can quantitatively indicate the degree of pollution and potential ecological risk caused by single or multiple pollutants [34].

$$E_{r}^{i}=T_{r}\frac{C_{i}}{C_{o}}$$

$$PERI=\sum_{1}^{n}E_{r}^{i}$$

where $E_{r}^{i}$ represents the potential ecological risk posed by an individual metal and REE, PERI is the potential ecological risk of the environment posed by multiple metals and REE. $C_{i}$ is the heavy metal and REE sample concentration in the sample, and $C_{o}$ is the background concentration. T_r_ is indicative of the toxic response factor for a specific heavy metal, and REE is as follows: Pb (5), Zn (1), Cu (5), As (10), Cd (30), Hg (40), Cr (2), Co (5), Ni (5), Ag (0.5), Ti (1), Mn (1), Gd (5), Nd (2), and La (1). The adjusted grading standards for $E_{r}^{i}$ (five categories) and PERI (four categories) are presented in Table 2 [27,[34], [35], [36], [37]].

**Table 2:** Grades of $E_{r}^{i}$ and *PERI* values for heavy metal contamination (Hakanson, 1980).

$E_{r}^{i}$ value	Ecological risk of single metal	*PERI value*	Potential ecological risk of environment
$E_{r}^{i}$*< 40*	Low risk	*PERI < 15*0	Low risk
40 ≤ $E_{r}^{i}$ < 80	Moderate risk	150 ≤ *PERI* < 300	Moderate risk
80 ≤ $E_{r}^{i}$ < 160	Considerable risk	300 ≤ *PERI* < 600	Considerable risk
160 ≤ $E_{r}^{i}$ < 320	High risk	*PERI ≥* 600	Very high risk
$E_{r}^{i}$*≥* 320	Very high risk		

## 3 Results and discussion

### 3.1 Magnetic characterization of heavy metals in sediments

The magnetic susceptibility measurement values of the samples in Table 3 show that the sediment samples from Inner Ambon Bay and the rivers (Lantamal River, Waitonahitu River, Waiheru River, and PLN Poka River) had varied magnetic susceptibility values. Inner Ambon Bay (S01–S012) had magnetic susceptibility $\left(\chi_{LF}\right)$ values ranging from 9.77 to 98.41 × 10^−8^ m^3^/kg with an average of 26.39 × 10^−8^ m^3^/kg, and the rivers (KL01-KL09) had magnetic susceptibility values ranging from 6.64 to 28.57 × 10^−8^ m^3^/kg with an average of 17.94 × 10^−8^ m^3^/kg. According to the information presented, the average magnetic susceptibility values found in IAB were higher than in the river. The KL01 sampling point in the PLN Poka River had the lowest susceptibility value $\left(\chi_{LF}\right)$ at 6.64 × 10^−8^ m^3^/kg, and the highest was at point S04 in Inner Ambon Bay, which is 98.41 × 10^−8^ m^3^/kg. The frequency-dependent magnetic susceptibility $(\chi_{FD\%}$) values in Inner Ambon Bay also ranged from 0.62% to 3.47%, with an average of 1.94%, and the rivers had $\chi_{FD\%}$ values ranging from 1.81% to 4.26%, with an average of 2.78%.

**Table 3:** Results of XRF analysis, which included the elements of Pb, Zn, Cu, As, Hg, Cr, Co, Ni, Ag, Ti, Mn, Fe, Gd, Nd, and La (mass %), as well as their low frequency magnetic susceptibility $\chi_{LF}$ (x 10^−8^ m^3^/kg), and frequency dependent magnetic susceptibility $\chi_{FD}$ (%).

Sampling Points	Heavy Metal Concentration												REEs			$\chi_{LF}$	$\chi_{FD}$
	Pb	Zn	Cu	As	Hg	Cr	Co	Ni	Ag	Ti	Mn	Fe	Gd	Nd	La		
S01	0.77	26.26	0.36	ND	0,22	0.04	0.37	0.11	0.52	0.59	0.11	16.22	0.09	0.09	0.22	15.58	2.07
S02	4.53	10.42	0.85	0.29	1.06	0.06	0.57	0.20	0.62	0.41	0.10	23.75	0.07	0.21	0.30	12.20	1.98
S03	1.33	9.82	0.58	ND	0.42	0.05	0.42	0.25	0.86	0.19	0.25	15.28	0.04	0.29	0.18	30.20	1.70
S04	1.77	11.06	0.82	0.44	0.50	0.13	0.78	0.82	0.90	0.56	0.34	30.62	0.07	0.26	0.21	98.41	2.96
S05	1.62	11.48	0.89	0.34	0.96	0.06	0.62	0.20	0.89	0.47	0.19	25.15	0.06	0.13	0.32	17.17	2.85
S06	1.63	12.13	0.68	0.93	0.41	0.07	0.40	0.28	0.76	0.25	0.41	14.96	0.21	0.18	0.23	40.38	2.31
S07	3.85	9.79	0.66	**22.70**	0.98	0.07	0.48	0.17	0.68	0.44	0.26	24.96	0.20	0.17	0.30	31.57	3.47
S08	2.65	9.07	0.62	ND	0.76	0.08	0.36	0.37	0.82	0.36	0.14	21.87	0.05	0.15	0.28	18.66	0.62
S09	3.77	10.60	0.81	ND	0.83	0.05	0.50	0.30	0.80	0.55	0.14	25.49	ND	0.20	0.25	14.04	0.95
S10	**5.97**	10.79	0.56	0.79	**1.48**	0.03	0.49	0.18	0.92	0.35	0.16	26.72	0.10	0.25	0.31	13.34	1.51
S11	4.04	10.10	0.68	0.95	1.03	0.07	0.35	0.13	0.89	0.44	0.13	22.08	0.13	0.11	0.25	9.77	0.88
S12	2.39	20.59	0.57	0.66	0.49	0.04	0.28	0.21	0.69	0.38	0.10	20.18	0.07	0.09	0.22	15.08	1.99
KL01	**7.58**	14.29	0.88	0.81	**1.91**	0.06	0.68	0.20	0.50	0.28	0.07	32.39	0.10	0.17	0.27	6.64	3.59
KL02	0.14	18.39	0.30	1.12	0.09	0.04	0.24	0.14	0.86	0.24	0.17	12.79	0.11	0.18	0.28	24.98	1.96
KL03	0.32	15.38	0.63	1.58	0.07	0.05	0.62	0.21	1.10	0.27	0.42	20.48	0.11	0.15	0.19	14.63	4.12
KL04	0.25	9.40	2.84	0.72	0.03	0.06	0.38	0.13	0.96	0.41	0.31	19.12	ND	0.17	0.24	16.32	1.81
KL05	0.15	19.02	0.56	0.51	0.02	0.04	0.32	0.15	0.72	0.72	0.19	12.18	0.09	0.10	0.23	11.36	2.01
KL06	2.67	10.67	0.69	0.42	0.16	0.06	0.38	0.23	1.02	0.34	0.23	21.20	0.13	0.23	0.22	15.15	2.36
KL08	2.65	9.07	0.62	ND	0.76	0.08	0.36	0.37	0.82	0.36	0.14	21.87	ND	0.15	0.28	25.85	4.26
KL09	3.77	10.60	0.81	ND	0.83	0.05	0.50	0.30	0.80	0.55	0.14	25.49	0.05	0.20	0.25	28.57	2.94
Average	2.59	12.95	0.77	2.30	0.65	0.06	0.46	0.25	0.81	0.41	0.20	21.46	0.10	0.17	0.25	23.00	2.32
Min	0.14	9.07	0.30	0.29	0.02	0.03	0.24	0.11	0.50	0.19	0.07	12.18	0.04	0.09	0.18	6.64	0.62
Max	7.58	26.26	2.84	22.70	1.91	0.13	0.78	0.82	1.10	0.72	0.42	32.39	0.21	0.29	0.32	98.41	4.26

Notes: ND means not detected or less than 0.01 %, S01 to S12 are sediment samples collected from IAB, KL01 to KL09 are samples collected from the rivers.

### 3.2 Assessment of the geoaccumulation index (I_geo_)

**Fig. 3:**
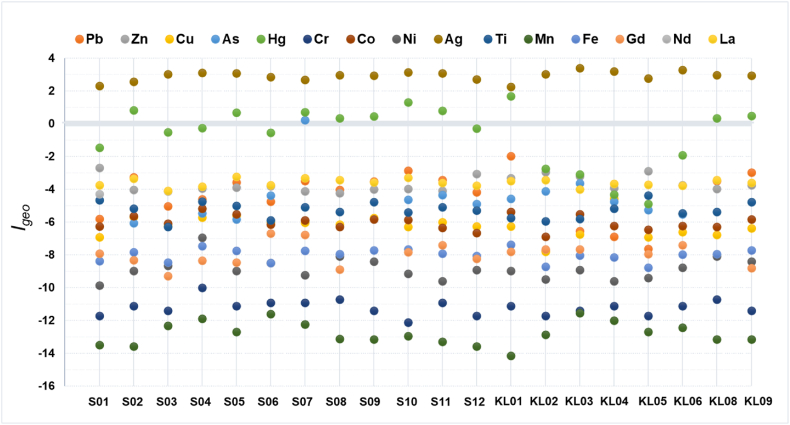
The geoaccumulation index (*Igeo*) of toxic heavy metals and REE in Inner Ambon Bay (S01–S12) and the rivers (KL01-KL09): *Igeo* ≤ 0 (uncontaminated), 0 < *Igeo* < 1 (uncontaminated to moderately contaminated), 1 < *Igeo* < 2 (moderately contaminated), 2 < *Igeo* < 3 (moderately to heavily contaminated), and 3 < *Igeo* < 4 (heavily contaminated).

The geoaccumulation index (*I*_*geo*_) values of heavy metals and rare earth elements (REE) in the surface sediments of Inner Ambon Bay and the rivers were calculated in Table 4, and Fig. 3 shows the indexes of sediment samples that were contaminated because of geoaccumulation. Ag, Hg, and As, based on the geoaccumulation index, were shown to be sources of pollution in Inner Ambon Bay and the surrounding rivers. The conditions in Inner Ambon Bay at sample point S07 ranged from uncontaminated to moderately contaminated by As, as classified in Table 1. The number of Inner Ambon Bays and rivers affected by Hg was 10, with 8 points (S02, S05, S07, S08, S09, S11, KL08, KL09) and 2 points (S10, KL01) of Inner Ambon Bay and rivers being uncontaminated to moderately contaminated. Sample point S08 in Inner Ambon Bay experienced the lowest concentration of contamination by Hg, while the highest contamination by Hg was at sample point KL01 in the PLN Poka River (moderately contaminated). Meanwhile, the presence of Ag was very abundant and had a high geoaccumulation index value at all sampling points, with 20 sampling points in Inner Ambon Bay and rivers polluted by Ag, with 11 points (S01, S02, S06, S07, S08, S09, S12, KL01, KL05, KL08, KL09) and 9 points (S03, S04, S05, S10, S11, KL02, KL03, KL04, KL06) in Inner Ambon Bay and rivers being moderately to heavily contaminated. The contamination levels of other heavy metals and REE elements, namely Pb, Zn, Cu, Cr, Co, Ni, Ti, Mn, Fe, Gd, Nd, and La, were all below zero, indicating that they were uncontaminated. The previous research conducted in the Milic Wetland area of Samsum, Turkey, also indicated that the Geoaccumulation Index values were found to be < 0, signifying that the sediments remained uncontaminated. Specifically, the values for As (−3.80), Zn (−3.34), Al (−3.33), Ni (−3.24), Cr (−3.06), Mn (−2.95), Fe (−2.91), Co (−2.87), Cu (−2.42), Pb (−1.88), and Cd (−1.88) to (−1.35) all fell below zero, indicating an absence of contamination for potentially hazardous elements (PHEs) in the sediment in that area [29].

**Table 4:** The geoaccumulation index (*Igeo*) to evaluate heavy metal and REE pollution in sediments.

Sampling Point	*Igeo*														
	Pb	Zn	Cu	As	Hg	Cr	Co	Ni	Ag	Ti	Mn	Fe	Gd	Nd	La
S01	−5.82	−2.70	−6.92	ND	−1.47	−11.72	−6.27	−9.86	2.32	−4.67	−13.51	−8.38	−7.91	−5.41	−3.67
S02	−3.26	−4.04	−5.68	−6.07	0.82	−11.14	−5.64	−8.99	2.57	−5.19	−13.58	−7.83	−8.34	−4.22	−3.35
S03	−5.03	−4.12	−6.23	ND	−0.51	−11.40	−6.08	−8.67	3.03	−6.30	−12.33	−8.47	−9.29	−3.72	−4.08
S04	−4.62	−3.95	−5.73	−5.47	−0.27	−10.02	−5.19	−6.96	3.10	−4.74	−11.89	−7.46	−8.36	−3.89	−3.83
S05	−3.59	−3.90	−5.62	−5.84	0.68	−11.14	−5.52	−8.99	3.09	−5.00	−12.70	−7.75	−8.47	−4.95	−3.25
S06	−4.74	−3.82	−6.00	−4.39	−0.56	−10.91	−6.15	−8.51	2.86	−5.91	−11.62	−8.50	−6.71	−4.46	−3.75
S07	−3.50	−4.13	−6.05	0.22	0.71	−10.91	−5.89	−9.23	2.69	−5.09	−12.24	−7.76	−6.77	−4.52	−3.31
S08	−4.04	−4.24	−6.14	ND	0.34	−10.72	−6.31	−8.11	2.96	−5.38	−13.13	−7.95	−8.89	−4.66	−3.44
S09	−3.53	−4.01	−5.75	ND	0.46	−11.40	−5.83	−8.41	2.94	−4.77	−13.15	−7.73	ND	−4.31	−3.59
S10	−2.87	−3.99	−6.28	−4.63	1.30	−12.14	−5.86	−9.15	3.13	−5.42	−12.95	−7.66	−7.85	−3.96	−3.29
S11	−3.43	−4.08	−6.00	−4.36	0.78	−10.91	−6.35	−9.62	3.08	−5.09	−13.29	−7.93	−7.41	−5.08	−3.60
S12	−4.18	−3.05	−6.26	−4.88	−0.29	−11.72	−6.67	−8.92	2.72	−5.30	−13.58	−8.06	−8.24	−5.47	−3.78
KL01	−1.98	−3.32	−6.26	−4.59	1.67	−11.14	−5.39	−8.99	2.25	−5.74	−14.15	−7.38	−7.80	−4.51	−3.49
KL02	−7.74	−2.95	−7.81	−4.12	−2.74	−11.72	−6.89	−9.51	3.03	−5.97	−12.87	−8.72	−7.66	−4.43	−3.44
KL03	−6.55	−3.21	−6.74	−3.63	−3.10	−11.40	−5.52	−8.92	3.39	−5.80	−11.57	−8.04	−7.66	−4.69	−4.00
KL04	−6.91	−3.92	−4.57	−4.76	−4.32	−11.14	−6.23	−9.62	3.19	−5.19	−12.01	−8.14	ND	−4.51	−3.66
KL05	−7.64	−2.91	−6.91	−5.26	−4.91	−11.72	−6.48	−9.41	2.78	−4.38	−12.71	−8.79	−7.95	−5.28	−3.72
KL06	−5.48	−3.74	−6.61	−5.54	−1.91	−11.14	−6.23	−8.79	3.28	−5.46	−12.44	−7.99	−7.42	−4.08	−3.78
KL08	−3.50	−3.97	−6.77	ND	0.34	−10.72	−6.31	−8.11	2.97	−5.38	−13.15	−7.95	ND	−4.69	−3.44
KL09	−2.99	−3.75	−6.38	ND	0.47	−11.40	−5.83	−8.41	2.93	−4.77	−13.15	−7.73	−8.80	−4.28	−3.60
Average	−4.57	−3.69	−6.24	−4.52	−0.62	−11.22	−6.03	−8.86	2.92	−5.28	−12.80	−8.01	−7.97	−4.56	−3.61
Min	−7.74	−4.24	−7.81	−6.07	−4.91	−12.14	−6.89	−9.86	2.25	−6.30	−14.15	−8.79	−9.29	−5.47	−4.08
Max	−1.98	−2.70	−4.57	0.22	1.67	−10.02	−5.19	−6.96	3.39	−4.38	−11.57	−7.38	−6.71	−3.72	−3.25

Notes: ND means not detected or less than 0.01 %.

### 3.3 The correlation between magnetism parameters and heavy metals

**Fig. 4:**
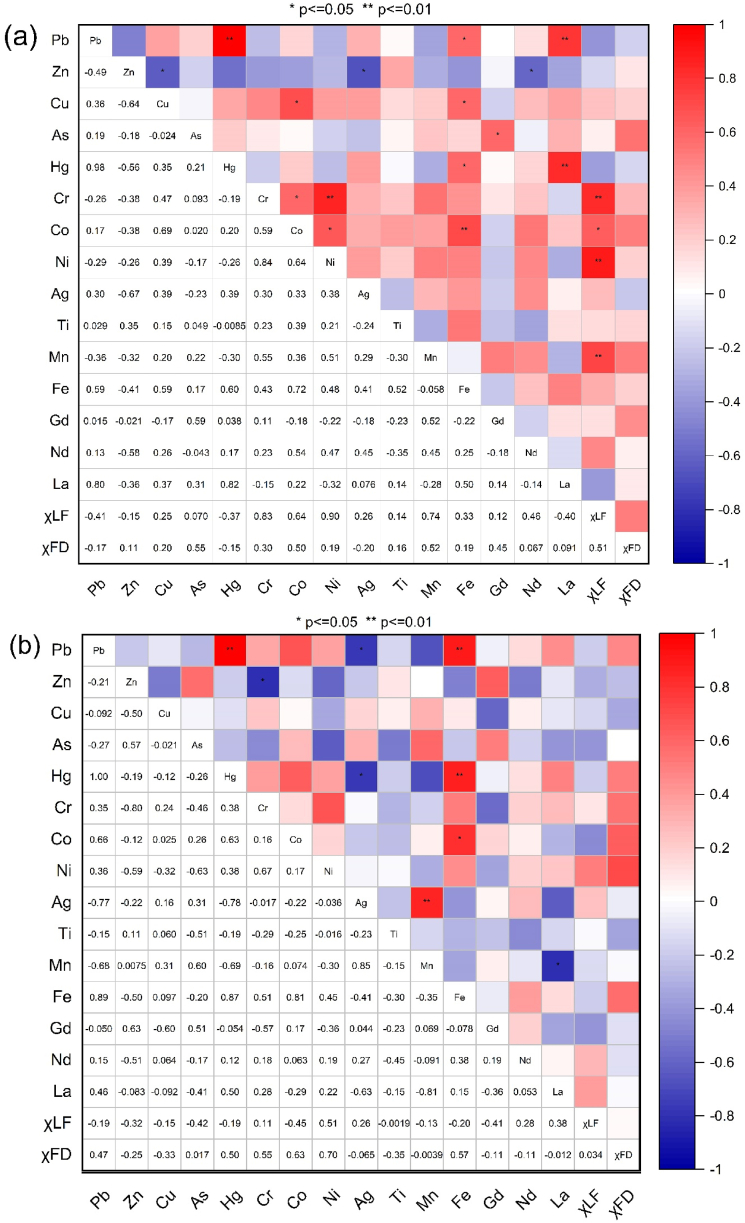
The correlation coefficients between the contents of heavy metals, REE, and magnetic susceptibility ($\chi_{\text{LF}}\;\text{and}\;\chi_{\text{FD}\%}$) (a) Inner Ambon Bay, (b) Lantamal River, Waitonahitu River, Waiheru River, and PLN Poka River.

The correlation coefficients involving magnetic parameters, heavy metal content, REE, and heavy metals and REEs themselves in sediment samples collected from Inner Ambon Bay and the surrounding rivers are presented in Fig. 4. These coefficients reveal the intensity of the connections between these parameters and provide an understanding of the level of interdependence among magnetic parameters. Within IAB (Fig. 4a), where 12 sampling points were considered, the Pearson correlation coefficients (*R*) between $\chi_{LF}$ and heavy metal concentrations ranged from *R* = 0.83 (for Cr) to *R* = 0.90 (for Ni), and also included *R* = 0.64 (for Co) and *R* = 0.74 (for Mn). The concentrations of the heavy metals Cr, Ni, and Co exhibited a strong correlation with $\chi_{LF}$, whereas the concentration of Mn showed a strong correlation with $\chi_{LF}$. This Pearson correlation analysis revealed that the significant correlation values of $\chi_{LF}$ found in the sediment case study inside Inner Ambon Bay supported its appropriateness as a reliable proxy indicator for determining Cr, Co, Ni, and Mn metal contamination.

Furthermore, there was a significant positive correlation between various pairs of heavy metals in both the Inner Ambon Bay (Fig. 4a) and the Rivers (Fig. 4b). Specifically, the heavy metal pairings Fe–Co exhibited a correlation coefficient of 0.72 in the Inner Ambon Bay and 0.81 in the Rivers. Similarly, the heavy metal pair Ni–Co showed a correlation coefficient of 0.64 in the Inner Ambon Bay, while the Cr–Ni pair exhibited a correlation coefficient of 0.84 in the same location. This suggests that these metals are elements with similar properties, thereby explaining the significant association. Group 8 elements on the periodic table of elements are also referred to as iron metals. In addition, there exists a highly significant correlation between the levels of mercury (Hg) and lead (Pb) in both the Inner Ambon Bay (0.98) and the rivers (1.00). The link between these two heavy metals can serve as an indicator of their toxicity in the environment. This verifies that there is a common occurrence of major mercury (Hg) contamination in conjunction with lead (Pb) contamination and vice versa.

### 3.4 Assessment of potential ecological risk of the environment

The potential ecological risk posed by an individual element ($E_{r}^{i})$ and multiple elements (*PERI*) in the Inner Ambon Bay and rivers is shown in Table 5. The $E_{r}^{i}$ values were in the order of Hg (65.00) > Ag (5.76) > As (1.77) > Pb (0.51) > Nd (0.13) > Zn (0.123) > Co (0.120) > Cu (0.11) > La (0.10) > Ti (0.04) > Gd (0.03) > Ni (0.02) > Fe (0.0060) > Cr (0.0013) > Mn (0.00024). On average, Hg presented a moderate ecological risk with an $E_{r}^{i}$ value of 65.00, and Ag, As, Pb, Nd, Zn, Co, Cu, La, Ti, Gd, Ni, Fe, Cr, and Mn presented a low ecological risk. The high $E_{r}^{i}$ Hg value (190.00) was found at sample point KL01 with the highest ecological risk, and the number of sampling points with considerable ecological risk and moderate risk by Hg were 7 (S10 > S02 > S11 > S07 > S05 > KL09 > S09) and 6 (S08 > KL08 > S04 > S12 > S03 > S06). The high $E_{r}^{i}$ Ag value (7.86) is located at sample point KL03 (low risk), whereas the high $E_{r}^{i}$ As value (17.46) is observed at sample point S07 (low risk), the high $E_{r}^{i}$ Pb value (1.90) was recorded at sample point KL01 (low risk), additionally followed by the $E_{r}^{i}$ contents with low risk of Nd, Zn, Co, Cu, La Ti, Gd, Ni, Fe, Cr, and Mn with values of 0.23 (S03), 0.23 (S01), 0.21 (S04), 0.32 (KL04), 0.12 (S05), 0.07 (KL05), 0.07 (S06), 0.06 (S04), 0.0090 (KL01), 0.0029 (S04), and 0.00049 (KL03).

**Table 5:** The potential ecological risk posed by an individual metal and REE ($E_{r}^{i})$ and the potential ecological risk of the environment posed by multiple metals and REEs (*PERI*).

Sampling Point	$E_{r}^{i}$ value															*PERI Index*	
	Pb	Zn	Cu	As	Hg	Cr (x10^−2^)	Co	Ni	Ag	Ti	Mn (x10^−3^)	Fe (x10^−2^	Gd	Nd	La		
S01	0.13	0.23	0.06	ND	21.67	0.09	0.10	0.01	3.75	0.06	0.13	0.45	0.03	0.07	0.09	26.20	Low Risk
S02	0.78	0.09	0.15	0.22	**105.72**	0.13	0.15	0.01	4.46	0.04	0.12	0.66	0.02	0.16	0.12	111.93	Low Risk
S03	0.23	0.09	0.10	ND	**42.22**	0.11	0.11	0.02	6.11	0.02	0.29	0.42	0.01	0.23	0.07	49.21	Low Risk
S04	0.31	0.10	0.14	0.34	**49.77**	0.29	0.21	0.06	6.43	0.06	0.40	0.85	0.02	0.20	0.08	57.72	Low Risk
S05	0.62	0.10	0.15	0.26	**96.13**	0.13	0.16	0.01	6.39	0.05	0.23	0.70	0.02	0.10	0.12	104.14	Low Risk
S06	0.28	0.11	0.12	0.72	**40.70**	0.16	0.11	0.02	5.44	0.03	0.48	0.42	0.07	0.14	0.09	47.82	Low Risk
S07	0.66	0.09	0.11	17.46	**97.86**	0.16	0.13	0.01	4.84	0.04	0.31	0.69	0.06	0.13	0.12	121.53	Low Risk
S08	0.46	0.08	0.11	ND	**76.08**	0.18	0.09	0.03	5.83	0.04	0.17	0.61	0.02	0.12	0.11	82.97	Low Risk
S09	0.65	0.09	0.14	ND	**82.67**	0.11	0.13	0.02	5.75	0.06	0.17	0.71	ND	0.15	0.10	89.77	Low Risk
S10	1.03	0.09	0.09	0.61	**147.87**	0.07	0.13	0.01	6.56	0.03	0.19	0.74	0.03	0.19	0.12	**156.79**	Moderate Risk
S11	0.70	0.09	0.12	0.73	**103.29**	0.16	0.09	0.01	6.35	0.04	0.15	0.61	0.04	0.09	0.10	111.65	Low Risk
S12	0.41	0.18	0.10	0.51	**48.95**	0.09	0.07	0.02	4.94	0.04	0.12	0.56	0.02	0.06	0.09	55.40	Low Risk
KL01	1.90	0.15	0.09	0.62	**191.00**	0.13	0.18	0.01	3.57	0.02	0.08	0.90	0.03	0.13	0.10	**197.84**	Moderate Risk
KL02	0.04	0.19	0.03	0.86	9.00	0.09	0.06	0.01	6.14	0.02	0.20	0.36	0.03	0.14	0.11	16.65	Low Risk
KL03	0.08	0.16	0.07	1.22	7.00	0.11	0.16	0.02	7.86	0.03	0.49	0.57	0.03	0.12	0.07	16.82	Low Risk
KL04	0.06	0.10	0.32	0.55	3.00	0.13	0.10	0.01	6.86	0.04	0.36	0.53	ND	0.13	0.09	11.27	Low Risk
KL05	0.04	0.20	0.06	0.39	2.00	0.09	0.08	0.01	5.14	0.07	0.22	0.34	0.03	0.08	0.09	8.20	Low Risk
KL06	0.17	0.11	0.08	0.32	16.00	0.13	0.10	0.02	7.29	0.03	0.27	0.59	0.04	0.18	0.08	24.43	Low Risk
KL08	0.66	0.10	0.07	ND	**76.00**	0.18	0.09	0.03	5.86	0.04	0.16	0.61	ND	0.12	0.11	83.07	Low Risk
KL09	0.94	0.11	0.09	ND	**83.00**	0.11	0.13	0.02	5.71	0.06	0.16	0.71	0.02	0.15	0.10	90.34	Low Risk
Average	0.51	0.123	0.11	1.77	65.00	0.13	0.120	0.02	5.76	0.04	0.24	0.60	0.03	0.13	0.09	73.19	
Min	0.04	0.08	0.03	0.22	2.00	0.07	0.06	0.01	3.57	0.02	0.08	0.34	0.01	0.07	0.07	8.20	
Max	1.90	0.23	0.32	17.46	191.00	0.29	0.21	0.06	7.86	0.07	0.49	0.90	0.07	0.23	0.12	197.84	

Notes: ND means not detected or less than 0.01 %.

The calculated *PERI* values for heavy metals and REEs ranged from 8.20 to 197.84,

with a total of 20 sample points falling within this range, with an average value of 73.19, indicating a low potential ecological risk. There were 11 locations in Inner Ambon Bay (S01, S02, S03, S04, S05, S06, S07, S08, S09, S11, S12) and 7 locations in rivers (KL02, KL03, KL04, KL05, KL06, KL08, and KL09) with low potential ecological risk. Meanwhile, S10 (156.79) in Inner Ambon Bay and KL01 (197.84) in PLN Poka River (Fig. 2c) were locations with a moderate potential ecological risk (Fig. 7). Significant heavy metals and REEs were found in Inner Ambon Bay at Locations S10: Hg (147.87) > Ag (6.56) > As (0.61) > Pb (1.03) > Nd (0.19) > Co (0.13) > La (0.12) > Zn (0.09) > Cu (0.09) > Ti (0.03) > Gd (0.03) > Ni (0.01) > Fe (0.0074) > Cr (0.0007) > Mn (0.00019), and in PLN Poka River, KL01 = Hg (191.00) > Ag (3.57) > Pb (1.90) > As (0.62) > Co (0.18) > Zn (0.15) > Nd (0.13) > La (0.10) > Cu (0.09) > Gd (0.03) > Ti (0.02) > Ni (0.01) > Fe (0.0090) > Cr (0.0013) > Mn (0.00008).

**Fig. 5:**
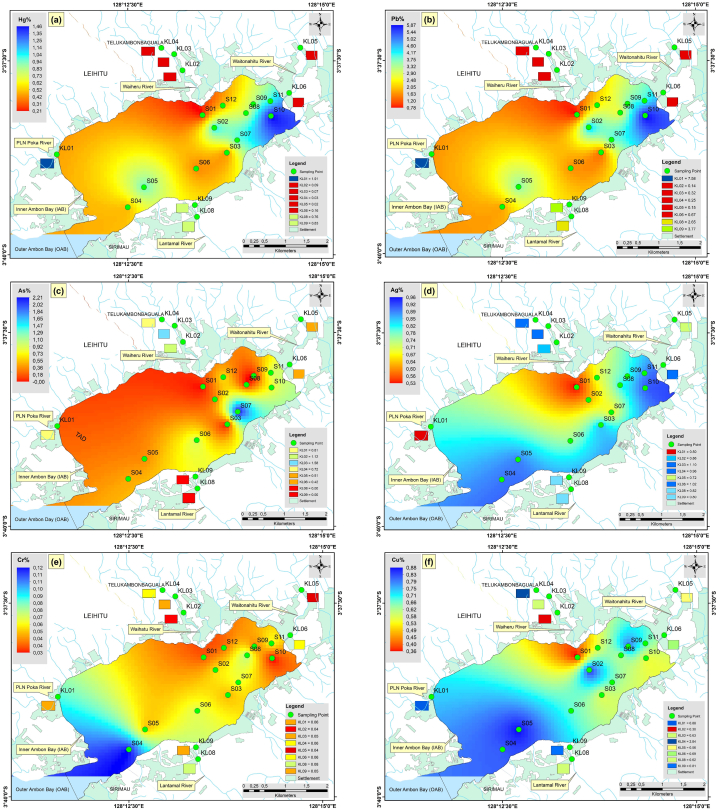

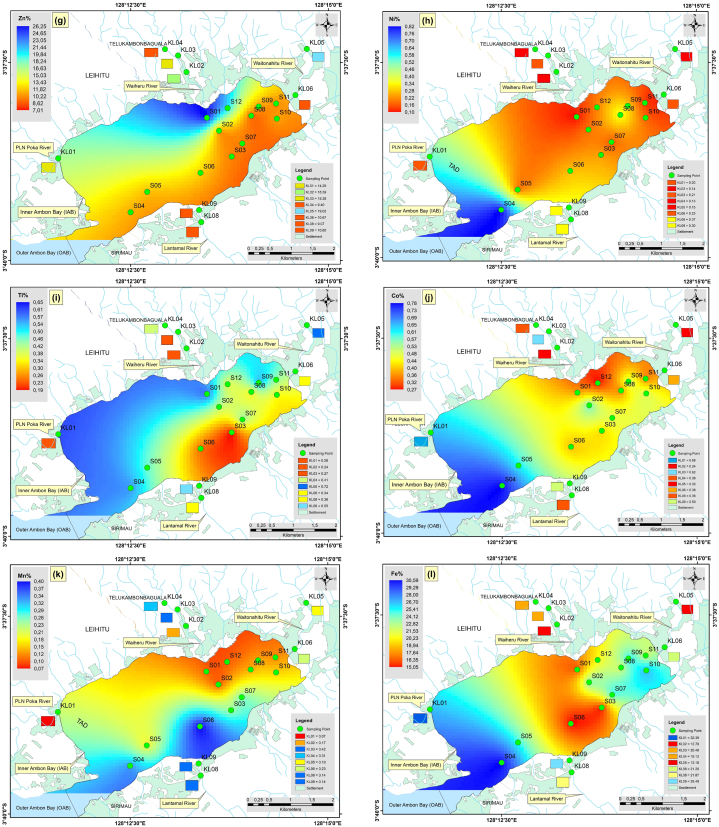
The spatial distribution of heavy metal contents in the surface sediments (a), Hg (b), Pb (c), As (d), Ag (e), Cr (f), Cu (g), Zn (h), Ni (i), Ti (j), Co (k), Mn (l), Fe

**Fig. 6:**
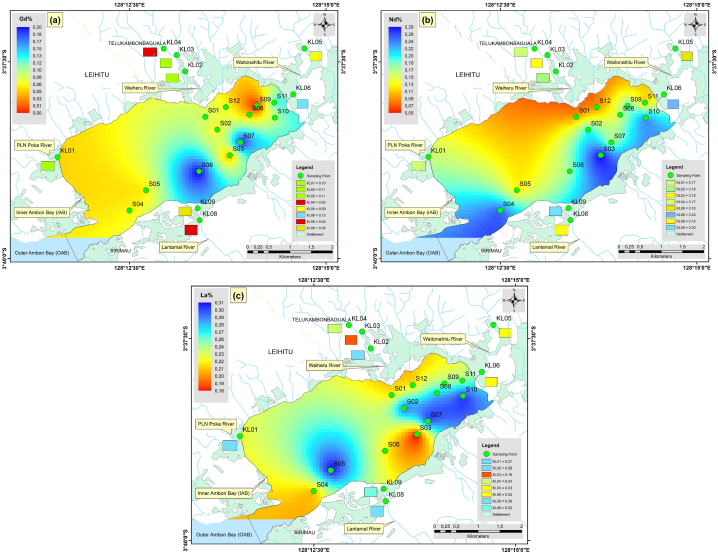
The spatial distribution of Rare Earth Elements in the surface sediments (a), Gadolinium (b), Neodymium (c), and Lanthanum.

**Fig. 7:**
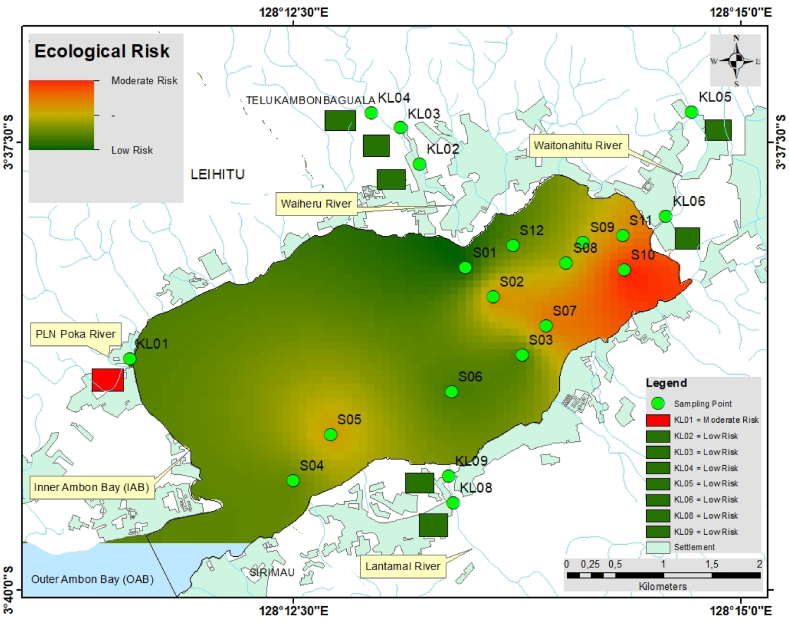
The spatial distribution of *PERI* represents the potential ecological risk posed by multiple metals and REE.

The evaluation results suggest that Hg is the primary pollution factor, with concentrations at points S10 and KL01 exceeding recommended limits. In contrast, Mn posed the lowest potential ecological risk. According to the guideline values for trace metals provided by the National Oceanic and Atmospheric Administration, USA [36], as well as the Canadian Sediment Quality Guidelines for the Protection of Aquatic Life [37], the threshold value for Hg (dry weight) is 0.71. However, the Hg values at S10 and KL01 are 1.48 and 1.91, which are above the trace metal threshold guideline value for Hg. This suggests that the potential risk factors are influenced not only by the level of heavy metal pollution but also by their inherent toxicity.

### 3.5 Spatial distribution of heavy metal and REE concentrations

The spatial distribution of the heavy metals is presented in Fig. 5, while that of REEs is presented in Fig. 6. Apart from that of Hg, the distributions of Pb, Ag, and As were also significant. In Fig. 5b, the distribution of Pb was significant in the eastern part at point S10 and the western part at point KL01. The threshold values for Pb were 8.0. However, the Pb values at S10 and KL01 are 5.97 and 7.58, respectively, so they are still below the guideline values for trace metal thresholds. Fig. 5c shows that the highest concentration of As was found in the western part of inner Ambon Bay at point S07, with a value of 22.70. This concentration is still below the threshold values for trace metals, set at 70 dry weight percent for As [38,39]. The distribution of Ag was significant at all sample points, except in the western part at point KL01 and the eastern part at point S01, which has a low concentration. The distribution of Ag concentration ranged from 0.50 to 1.10 with an average of 0.81; this value is also still below the threshold guideline value for the trace metal limit for Ag, namely 3.7 dry weight. There was something interesting about the distribution of Gd (Fig. 6a), where the concentration of Gd is high in the southern part at points S06 and S07, where these points were close to the hospital and health center. It can be assumed that the high concentration of Gd at these two points could be sourced from the river, which had been contaminated by anthropogenic Gd from hospitals [40]. Prior investigations have shown that sources of medical and urban waste contain Gd. In magnetic resonance imaging (MRI), Gd is frequently employed as a contrast agent. Stable Gd chelates may be transferred from health centers into municipal sewage systems together with antibiotics, antihypertensive drugs, anti-inflammatories, antihistamines, and estrogens, eventually ending up in rivers and marine environments [[41], [42], [43]]. Gd also weakened the positive correlation with a moderate level towards As with a value of 0.59 (Fig. 4a). The significant distribution of Nd (Fig. 6b) and La (Fig. 6c) at points S04 and S10 confirms their anthropogenic origin, primarily from human activities. The majority of these components were transported to the ocean through river runoff and eolian transport. The composition of dissolved REE is controlled by the stability of complex compounds, while the chemistry of suspended REE in river waters reflects the rock composition of drainage areas [44,45].

### 3.6 The relationship between MS and heavy metal content and source

The measurement of $\chi_{LF}$ in this study has proven to provide valuable information about the level of pollution in Ambon Bay. This research demonstrates that $\chi_{LF}$ showed a strong correlation with heavy metals such as Cr, Co, Ni, and Mn, in locations that tend to be less polluted, like S01, S02, S03, S04, S05, S06, S07, S08, S09, S11, S12, KL02, KL03, KL04, KL05, KL06, KL08, and KL09. At points S10, with a $\chi_{LF}$ value of 13.34 × 10^−8^ m^3^/kg, and point KL01, with a $\chi_{LF}$ value of 6.64 × 10^−8^ m^3^/kg, heavy metal pollution was evident, even though the $\chi_{LF}$ values are low. This is due to the presence of Hg, Zn, and Cu, which have a negative correlation with $\chi_{LF}$, as found in Kalimantan Selatan, Indonesia [9]. In this study, it was also found that high values of Hg, Zn, and Cu exhibit an inverse relationship with low $\chi_{LF}$ values but have a direct or positive correlation with $\chi_{FD\%\;}$. The presence of Hg contamination correlated to an increase in χ_FD%_ values at specific sample locations, i.e., points S10 (1.51%) and KL01 (3.59%).

Sampling point S10 in Inner Ambon Bay, with $\chi_{LF}$ values of 13.34 × 10^−8^ m^3^/kg exhibiting moderate potential ecological risk, was influenced by anthropogenic processes originating from emissions, corrosion, and erosion from machinery and motor vehicles, as well as household waste [46]. The economic activities and the presence of numerous floating fisheries, shipwrecks, and vessels in Ambon Bay also contribute to pollution through the presence of magnetic minerals in Inner Ambon Bay, such as the use of anti-rust paint on ship hulls releasing metals Co and Zn [37]. The presence of As (Fig. 5c) at location point S07 can be related to agricultural activities due to the use of herbicides, insecticides, and fertilizers containing inorganic As [47]. The heavy metal content of Pb in Inner Ambon Bay can also originate from aerosol deposition, such as the deposition of ship exhaust released into the air and settling into the sea; therefore, reducing emissions from ship exhaust is one way to mitigate the adverse effects of this detrimental aerosol deposition [48]. Pb can come from both natural and industrial sources. For example, aragonite, which is abundant in Pb and Zn, is found in the primary bedrock near Mahikeng [49]. Significant levels of Hg, As, and other heavy metals are present in wastewater and other industrial processes, and these are the main sources of soil heavy metal pollution [50]. On the other hand, the significant presence of heavy metals Hg and Pb, causing damage to mangrove forests as observed in location KL01 (Fig. 5a and. b), can be attributed to PLN (National Electricity Company) industrial waste. Reports from the local media have highlighted oil spills caused by pipeline leaks in the area [51]. Additionally, damage to mangrove forests in Staten Island, New York City, USA, attributed to the presence of Pb and Hg from oil spills, has also been documented [52]. While the presence of Ag (Fig. 5d) was highly significant in all samples, it may not originate from industry or household waste but rather from a lithogenic process. Considering the geological conditions of Ambon Island, which is composed of volcanic environments, Ag may originate from hypothermal and epithermal deposits formed in a volcanic environment, where underground hot water can transport these minerals to the surface. When this hot water reaches the surface, its mineral solution cools down and forms minerals such as argentite (Ag_2_S) along with other minerals. Further studies are required to investigate this possibility.

### 3.7 Contaminant source identification using multivariate statistical analysis and assessment of contamination origin

**Fig. 8:**
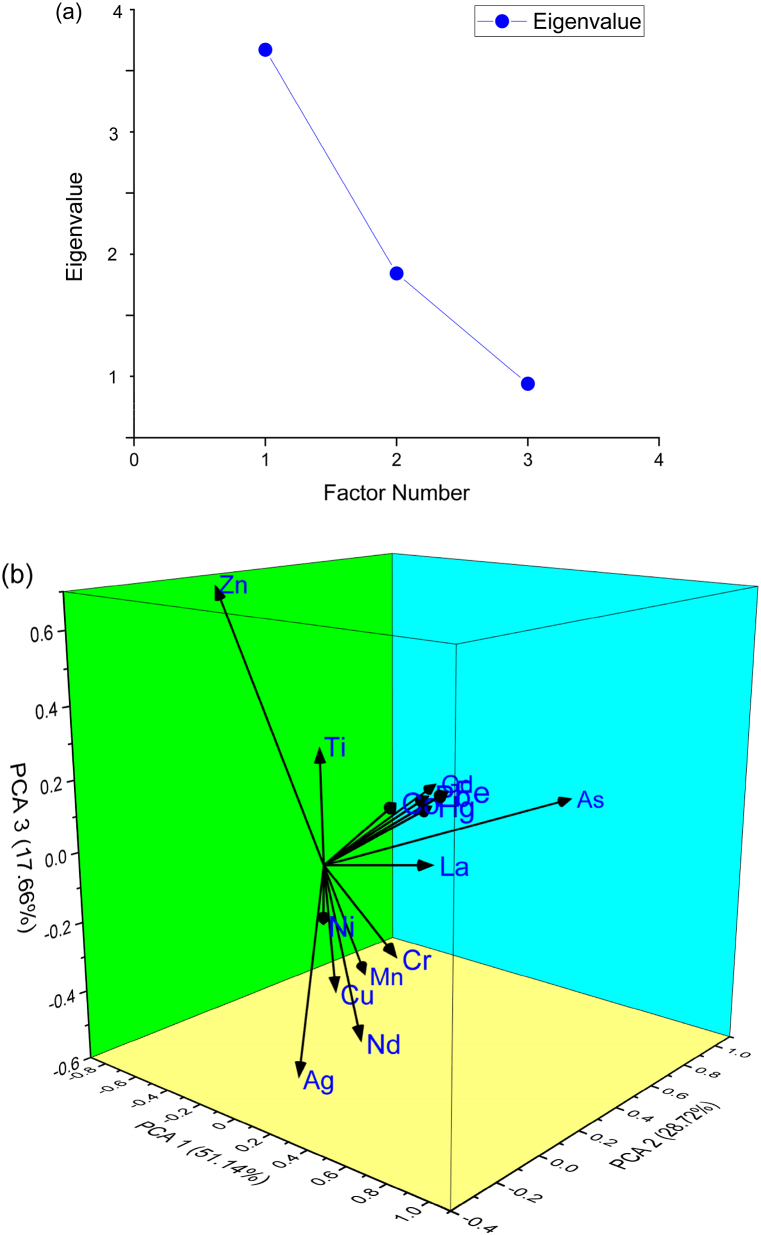
Loading plot of the PCA of trace toxic heavy metals in the Inner Ambon Bay and the rivers sediments. PCA of measured parameters by (a) scree plot of the characteristic roots (eigenvalues) and (b) component plot in rotated space.

Multivariate data analysis, especially PCA (Principal Component Analysis) and HCA (Hierarchical Cluster Analysis), is used to determine the source and amount of toxic heavy metals accumulated in Inner Ambon Bay (IAB) sediments. The sample data collected in this study is sufficient for PCA, as indicated by Bartlett's sphericity test (p < 0.001). PCA reveals three main components with eigenvalues greater than 1, accounting for 97.52% of the total variance. Therefore, component plots in the rotated space indicate that toxic heavy metals in sediment specimens are associated with three different sources (Fig. 8). In PCA1, metals (Ag, Mn, Nd, Cr, and Cu) contribute to 51.14% of the total variance. Conversely, PCA2 accounts for 28.72% of the total variation in terms of toxic heavy metals (As, La, Hg, Gd, Fe, and Pb). PCA3 contributes to 17.66% of the total variation, involving elements (Zn, Ti, Ni, and Co). PCA suggests that metals in PCA1 (Ag, Mn, Nd, Cr, and Cu) may originate from lithogenic inputs, while metals in PCA2 (As, La, Hg, Gd, Fe, and Pb) can be interpreted using both anthropogenic and lithogenic inputs. However, metals in PCA3 (Zn, Ti, Ni, and Co) are likely associated with anthropogenic sources.

**Fig. 9:**
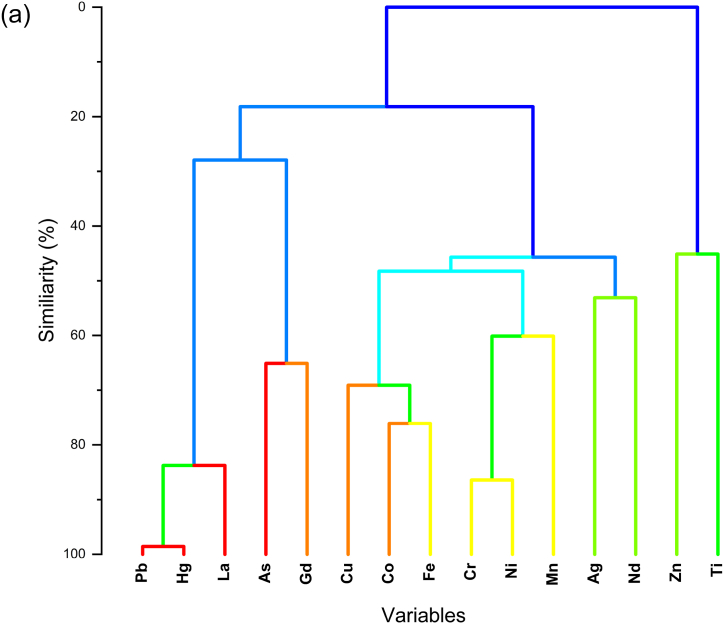
Dendogram for HCA of trace toxic heavy metals in sediments of Inner Ambon Bay.

Furthermore, further verification of PCA is performed using HCA. According to the results shown in Fig. 9, the PCA results align with the dendrogram of HCA, where the HCA dendrogram for trace toxic heavy metals in the sediments of Inner Ambon Bay reveals three distinct groups. The first group includes elements Pb, Hg, La, As, and Gd, while the second group consists of elements Cu, Co, Fe, Cr, Ni, Mn, Ag, and Nd. The elements forming the third group are Zn and Ti. These findings are consistent with the results of Igeo, PERI Index, and the initial hypothesis based on XRF analysis that identifies Pb, Hg, La, As, and Gd as contributors to anthropogenic sources. Considering the presence of oil spills at sample point KL01 and the use of anti-rust paint on ship hulls, these factors contribute significantly to anthropogenic contamination. Additionally, agricultural activities such as the use of fertilizers and pesticides, along with the input of hospital waste, are significant sources of anthropogenic pollution.

Fig. 4a shows the outcomes of the PCC test, highlighting the strong correlations among specific toxic heavy metals: Hg–Pb (r = 0.98), Pb–La (r = 0.80), Hg–La (r = 0.82), As–Gd (0.59), Ni–Co (0.64), Hg–Fe (0.60), La–Fe (r = 0.50), Fe–C0 (0.72), Pb–Fe (r = 0.59), and Mn–Cr (0.55). In this study, significant associations were identified among toxic heavy metals within identical clusters and principal components, indicating a cohesive reinforcement among the outcomes of PCA, HCA, and PCC. In another study conducted in the Çavuşlu River, Turkey, pollution impacts were observed, especially in the areas around the Garbage Disposal Facility (GDF). Consequently, preventive measures are necessary to reduce the risk of pollution and preserve environmental health in that region. The concentration of metals in sediments indicated low to moderate contamination levels. Specific metals, such as Ni, exhibit higher contamination levels, indicating a toxic response to benthic organisms. While most stations showed low pollution levels, stations around the GDF demonstrated higher pollution levels, particularly for certain heavy metals [29]. Our study similarly revealed that the majority of stations exhibited low pollution levels. However, stations around the sample points KL01 and S10 showed elevated pollution levels, requiring special attention.

The observed variations in heavy metal concentrations among different stations in Inner Ambon Bay and its surrounding rivers can be attributed to a combination of natural and anthropogenic factors shaping the environmental conditions. Anthropogenic activities, such as industrial discharges (PLN) and urban/agricultural runoff, contribute to elevated heavy metal levels in certain areas. Stations proximate to industrial zones may experience higher concentrations due to pollutants released into the water, while those closer to urban or agricultural areas may be affected by runoff carrying contaminants from streets and residential zones. Point sources of pollution, such as oil spills from shipping activities and improper waste disposal, also contribute to spatial disparities in heavy metal values. Natural factors, including the geological composition of sediments and riverine input, play a role in influencing concentrations. Additionally, spatial variability driven by currents, tidal patterns, and sediment characteristics contributes to differences in heavy metal distribution. Local anthropogenic sources, like specific industries in the vicinity of certain stations, further exacerbate variability. Overall, this complex interplay of factors underscores the need for comprehensive understanding and ongoing monitoring to effectively manage heavy metal contamination in coastal environments.

## 4 Conclusions

The distribution of heavy metals and the assessment of ecological risk levels in Ambon Bay have been successfully conducted. In the sampling locations S10 and KL01, relatively high concentrations of heavy metals and REEs were found, while sampling locations S01, S02, S03, S04, S05, S06, S07, S08, S09, S11, S12, KL02, KL03, KL04, KL05, KL06, KL08, and KL09 had relatively low levels of heavy metals and REEs. The calculated *Igeo* values indicated that the highest number of sampling locations were polluted by Ag, Hg, and As, while As had the least contamination. The $E_{r}^{i}$ values also showed that Hg posed the highest ecological risk due to its high toxicity factor, whereas Mn had the lowest potential ecological risk. The calculation of the Heavy Metal Pollution Index (*PERI*) values indicated that most locations in Ambon Bay were at low potential ecological risk, whereas S10 in IAB and KL01 in PLN Poka River were locations with a moderate potential ecological risk. The primary sources of heavy metals in Ambon Bay were anthropogenic processes such as household waste, oil spills from the national electricity company, the use of anti-rust paint on ship hulls, emissions, corrosion, and erosion from machinery and motor vehicles, all of which contribute to heavy metal pollution. Inner Ambon Bay had magnetic susceptibility $\left(\chi_{LF}\right)$ values ranging from 9.77 to 98.41 × 10^−8^ m^3^/kg with an average of 26.37 × 10^−8^ m^3^/kg. The concentrations of heavy metals in Ambon Bay, such as Cr, Ni, Co, and Mn, were significantly positively correlated with the $\chi_{LF}$ content. Therefore, $\chi_{LF}$ measurement techniques provide a cheaper and less time-consuming method for identifying potential contamination in the bay. Magnetic susceptibility ($\chi_{LF})$ measurements can be used as a proxy measure for the level of heavy metal contamination and reveal the distribution of polluted areas. Multivariate statistical analysis (principal component analysis (PCA), Pearson's correlation coefficient (PCC), and hierarchical cluster analysis (HCA)) outlined that the metallic accumulation in the sediments of IAB was related to lithological, geological, and anthropogenic impacts. This research can provide a scientific foundation for the environmental management of Inner Ambon Bay and similar fishing harbors. Subsequent studies may focus on conducting a more comprehensive analysis of household waste composition, examining the impact of anti-rust paint on ship hulls, and assessing the effects of pollutants, corrosion, and erosion from machinery and automobiles. Moreover, conducting an extensive investigation into the long-term consequences of heavy metal pollution on the bay's ecosystem and the potential bioaccumulation in marine organisms would generate crucial insights for implementing environmentally sustainable management practices. This proposed line of inquiry aligns with our mission to furnish empirically supported data for effective environmental management strategies in Inner Ambon Bay and other similar fishing harbors.

## Funding statement

This work was supported by 10.13039/501100015689Institut Teknologi Bandung (ITB) and the Indonesian Endowment Fund for Education Agency (10.13039/501100014538Lembaga Pengelola Dana Pendidikan, LPDP).

## Data availability statement

All the data are presented in the text except for bathymetric data and coordinates of sampling points. Bathymetric data and coordinates of sampling points can be found in the following link.

https://bit.ly/Noya-et-al-2024.

## Additional information

No additional information is available for this paper.

## CRediT authorship contribution statement

**Yohansli Noya:** Writing – review & editing, Writing – original draft, Visualization, Validation, Resources, Project administration, Methodology, Investigation, Funding acquisition, Formal analysis, Data curation, Conceptualization. **Satria Bijaksana:** Writing – review & editing, Writing – original draft, Visualization, Validation, Resources, Project administration, Methodology, Investigation, Funding acquisition, Formal analysis, Conceptualization. **Silvia Jannatul Fajar:** Writing – review & editing, Writing – original draft, Investigation, Data curation, Conceptualization. **Putu Billy Suryanata:** Writing – review & editing, Writing – original draft, Investigation. **Ulvienin Harlianti:** Writing – review & editing, Writing – original draft, Investigation. **Khalil Ibrahim:** Writing – review & editing, Writing – original draft, Visualization. **Ni Komang Tri Suandayani:** Writing – review & editing, Writing – original draft, Validation, Methodology. **Warni Multi:** Writing – review & editing, Writing – original draft, Investigation. **Samsul Bahri:** Writing – review & editing, Writing – original draft, Investigation.

## Declaration of competing interest

The authors declare that they have no known competing financial interests or personal relationships that could have appeared to influence the work reported in this paper.
